# Supplementary material for: Crystal structures of phosphatidyl serine synthase PSS reveal the catalytic mechanism of CDP-DAG alcohol O-phosphatidyl transferases
Source: Nat Commun. 2021 Nov 30;12:6982. doi: 10.1038/s41467-021-27281-w (PMC8633023; doi:10.1038/s41467-021-27281-w)
Supplement: Supplementary file 1 — Supplementary Information [file 41467_2021_27281_MOESM1_ESM.pdf]

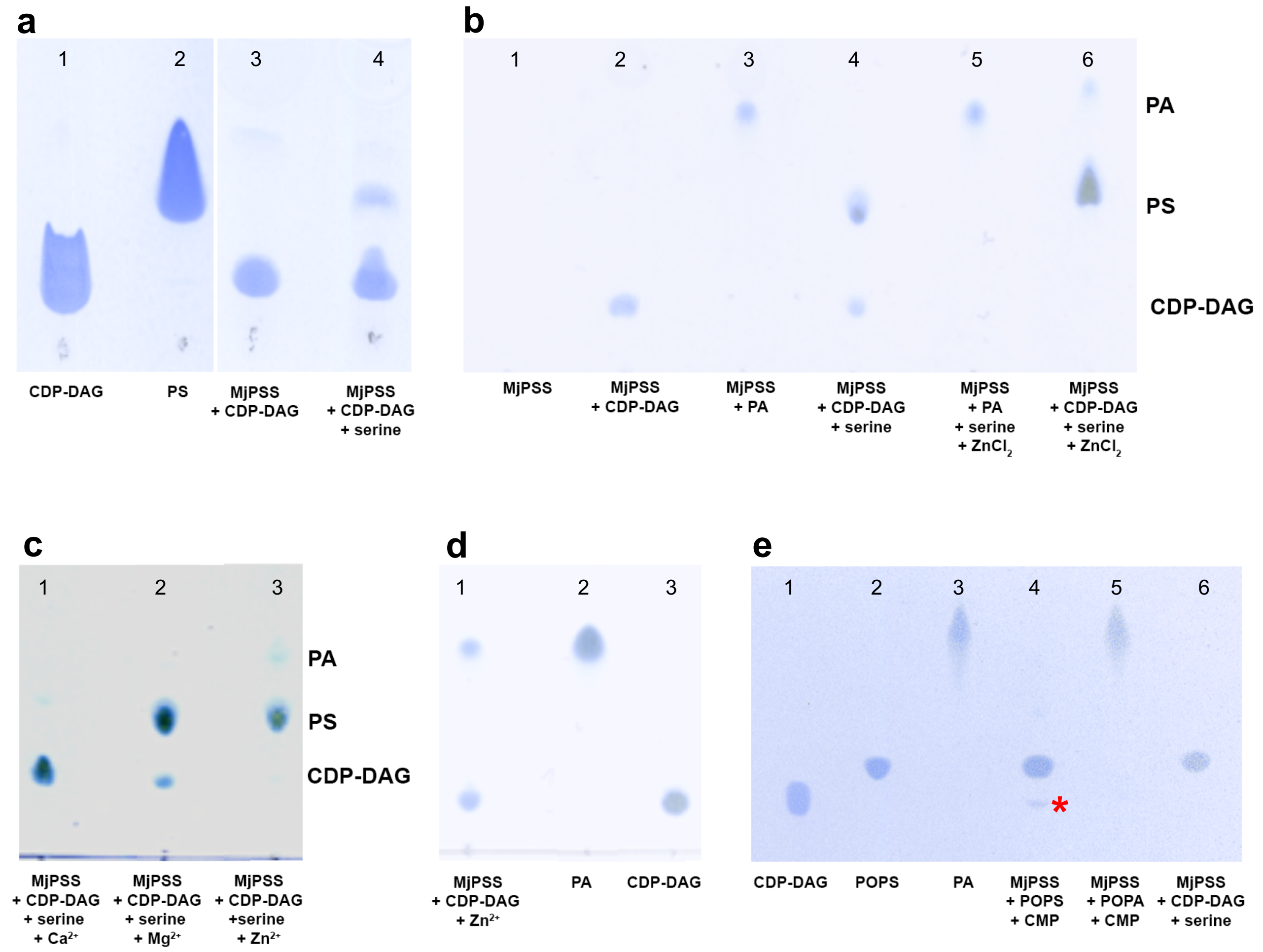

Supplementary Figure 1: Analysis of MjPSS activity by thin layer chromatography (TLC). a) TLC analysis of educts and products after incorporation of MjPSS in lipidic cubic phase. PS is synthetised by MjPSS only if both, CDP-DAG and serine are present in the reaction mixture (lane 4). In the absence of serine (lane 3) no reaction is observed with CDP-DAG. Synthetic CDP-DAG and PS are shown in lane 1 and 2 for reference. b) Analysis of the reaction products of MjPSS in presence of Zn^{2+}. No lipids were copurified with MjPSS (lane1). Incubation of MjPSS with CDP-DAG (lane 2) or PA (lane3) shows no reaction. PS is synthetized by MjPSS with CDP-DAG and serine (lane 4). In the presence of Zn^{2+}, PA is also formed in addition to PS (lane 6) and CDP-DAG fully converted. No reaction is observed when MjPSS is incubated with PA, serine and Zn^{2+} (lanes 5). c) TLC analysis shows that Ca^{2+} is unable to restore the inhibition by EDTA (lane 1), whereas recovery by Mg^{2+} is almost complete (lane 2). In the presence of Zn^{2+}, MjPSS hydrolyses CDP-DAG to PA and PS (lane3). d) MjPSS does not require serine for the hydrolysis of CDP-DAG to PA in the presence of Zn^{2+}. e) MjPSS is able to catalyse the reverse reaction when incubated with the products POPS and CMP (lane 4). CDP-DAG, the product of the reverse reaction, is highlighted by the red asterisk. MjPSS is unable to catalyse the formation of CDP-DAG from POPA and CMP (lane 5). In the presence of CDP-DAG and serine, MjPSS catalyses the reaction to PS (lane 6). In lanes 1-3, CDP-DAG, POPS, and POPA are shown as control.

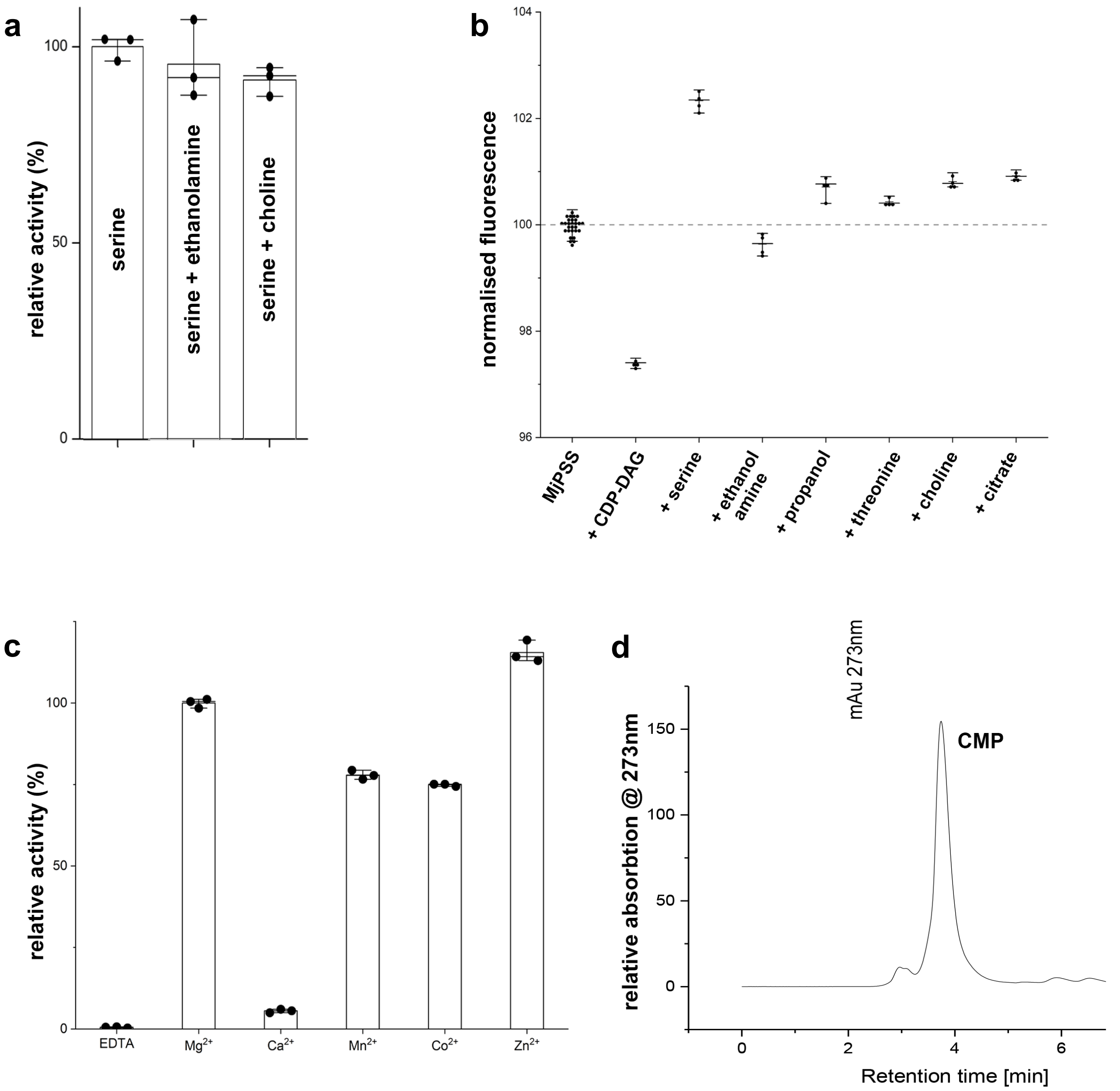

Supplementary Figure 2: Analysis of substrate binding and activity of MjPSS. a) MjPSS is highly selective for its substrate serine. The enzyme activity is barely reduced by incubation with serine in the presence of structurally similar alcohols such as ethanolamine or choline (n = 3; mean ± s.d.). b) Normalised fluorescence change of MjPSS in the presence of its substrates and substrate-like molecules (n = 24 for MjPSS; n=4 the rest; mean ± s.d.). c) The activity of MjPSS is inhibited by EDTA and is restored by additional Mg^{2+}, Mn^{2+}, Co^{2+} and Zn^{2+}, while additional Ca^{2+} has no effect. The restoration by Mn^{2+} and Co^{2+} does not reach the levels observed with Mg^{2+}, while a slight hyperactivation is observed with Zn^{2+} (n = 3; mean ± s.d.). d) HPLC analysis shows that CMP is also released by hydrolysis of CDP-DAG by MjPSS in the presence of Zn^{2+} and in the absence of serine.

a

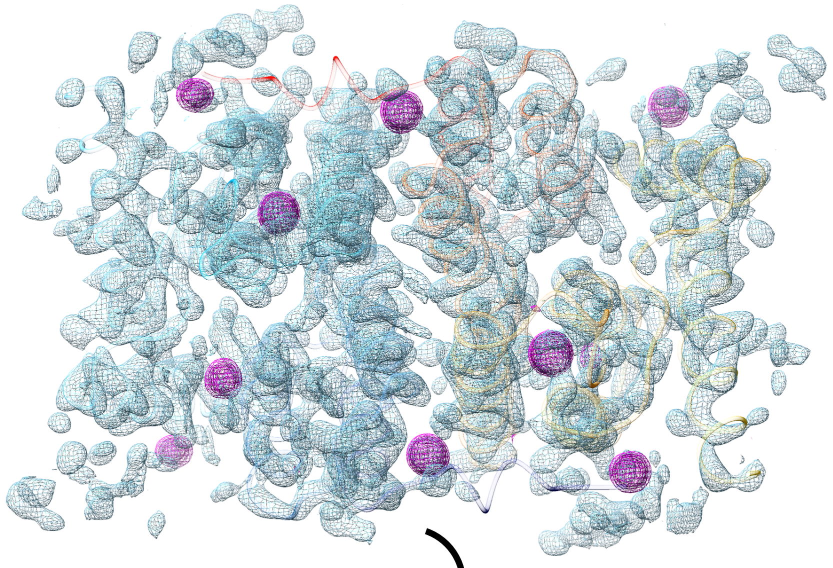

b

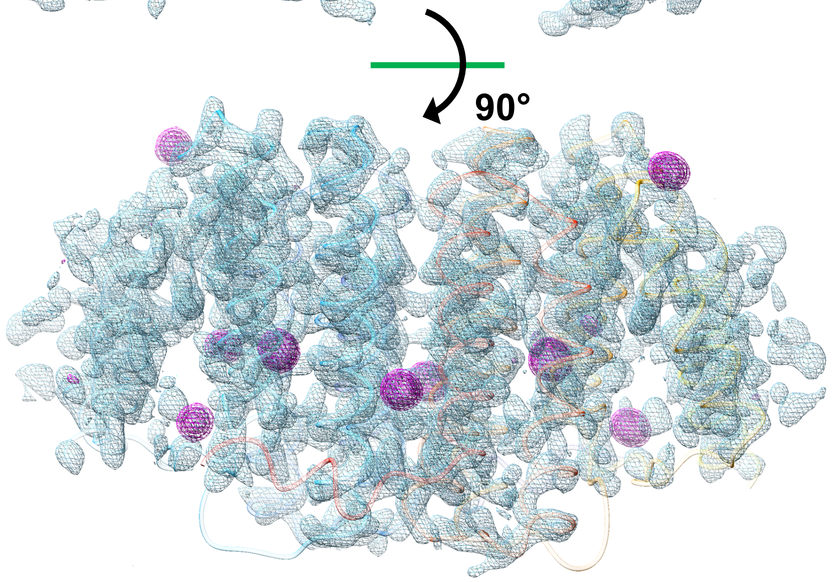

c

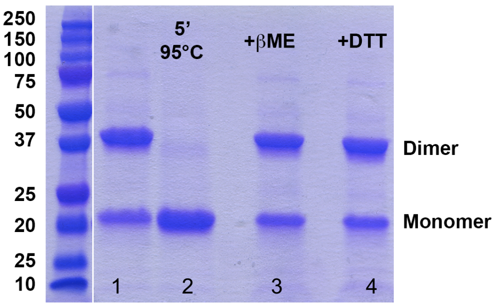

Supplementary Figure 3: Cytoplasmic (a) and side view (b) of the experimental electron density map for the MjPSS dimer after phasing and density modification. Strong density peaks in the anomalous difference map (magenta) indicate the positions of the selenium atoms. The final refined model is shown as blue ribbon for one protomer and red/yellow ribbon for the other. c) SDS-PAGE analysis shows that the detergent solubilized MjPSS is a SDS stable dimer (lane 1). The dimer monomerises after heating the sample to 95°C for 5 minutes (lane 2). The dimerisation is not mediated by disulfide bridges, as the addition of the reducing agents DTT or β-mercaptoethanol show no effect (lane 3 and 4). The experiments were performed for each purification (at least three times) with reproducible SDS-PAGE pattern.

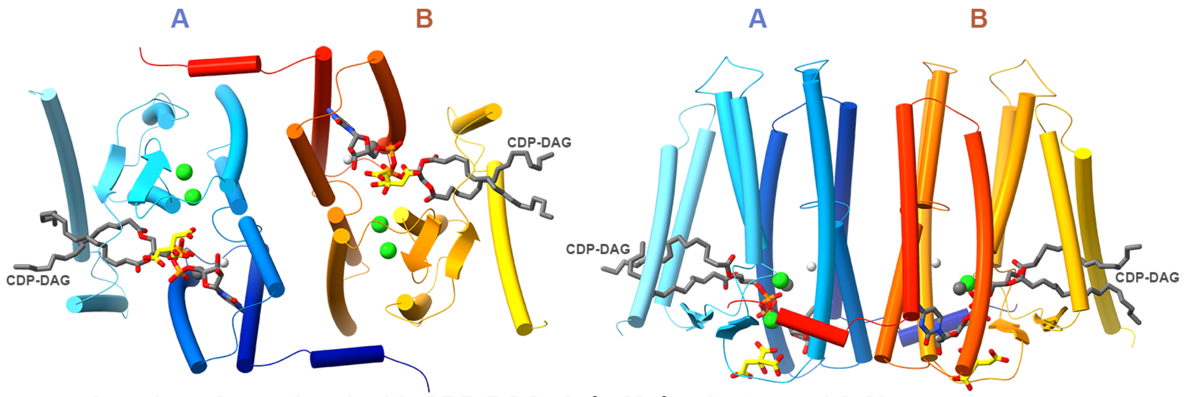

closed conformation 1 with CDP-DAG, Ca^{2+}, Mg^{2+}, citrate, and 2 Cl^- per monomer

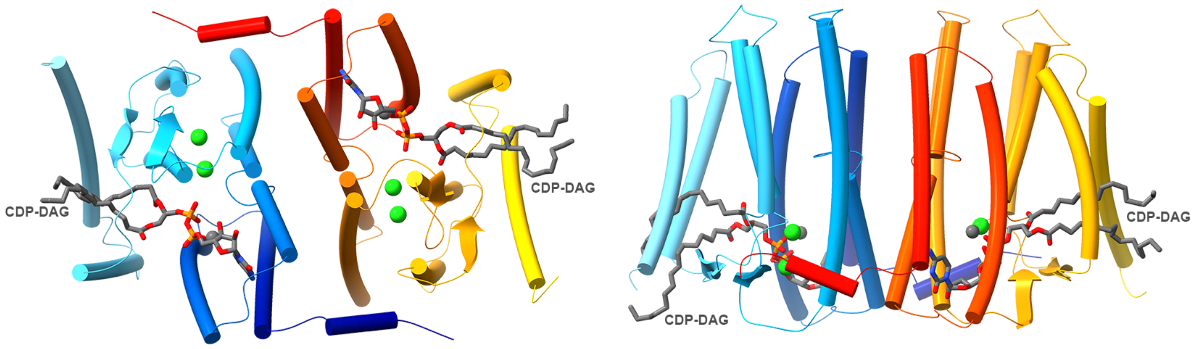

closed conformation 2 with CDP-DAG, Ca^{2+}, 2 Cl^- per monomer

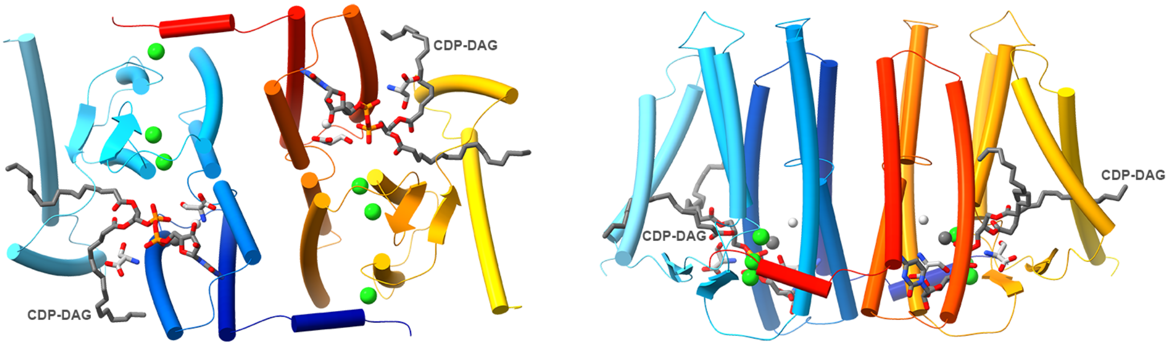

open conformation 1 with CDP-DAG, 2 serines, Ca^{2+}, Mg^{2+}, and 3 Cl^- per monomer

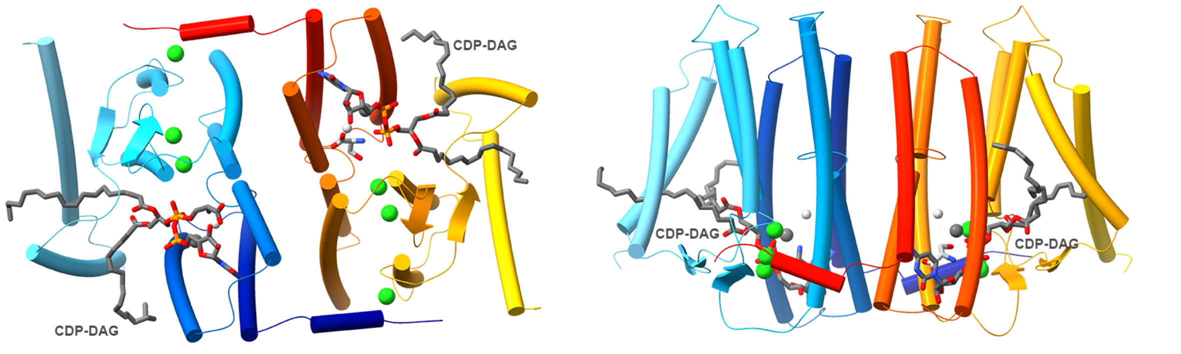

open conformation 2 with CDP-DAG/serine in transition state, Ca^{2+}, Mg^{2+}, and 3 Cl^- per monomer

Supplementary Figure 4: Overview of the obtained MjPSS structures in different states.

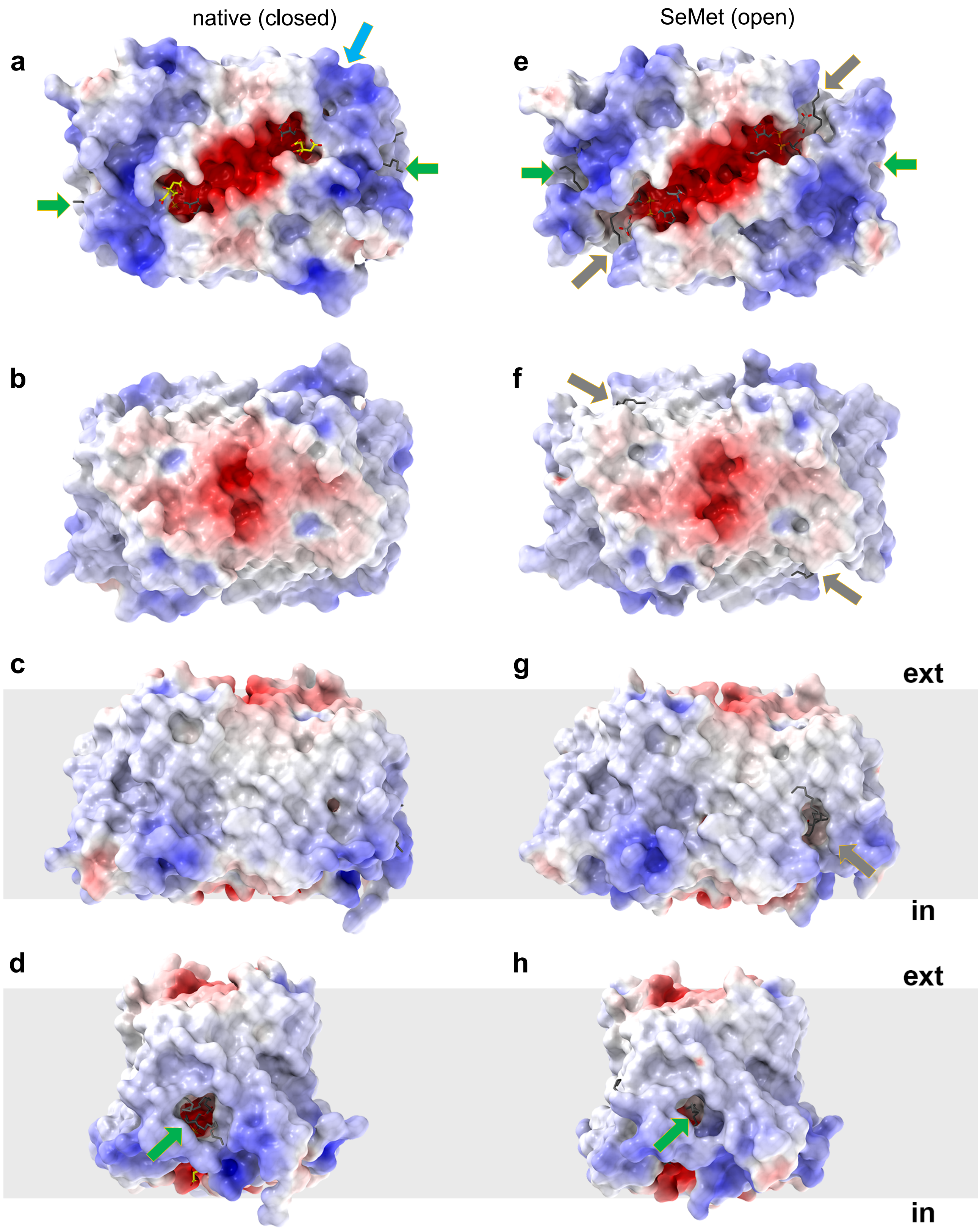

Supplementary Figure 5: Electrostatic surface potential and bound ligands. The substrate binding site on the cytoplasmic side of MjPSS in the closed conformation (a-d) with bound CDP-DAG (grey) and citrate (yellow) is strongly negatively charged (a). Negative charges also dominate on the outer side (b). While in the closed structure the negatively charged trench in the dimer centre is surrounded by positive charges, in the open structure (e-h) a gap (grey arrow) connects the trench with the hydrophobic membrane core (e, f, g). A hydrophobic channel (green arrow) connects the substrate binding site to the membrane core (a, d, e, h) and is occupied by the alkyl chain of the bound CDP-DAG.

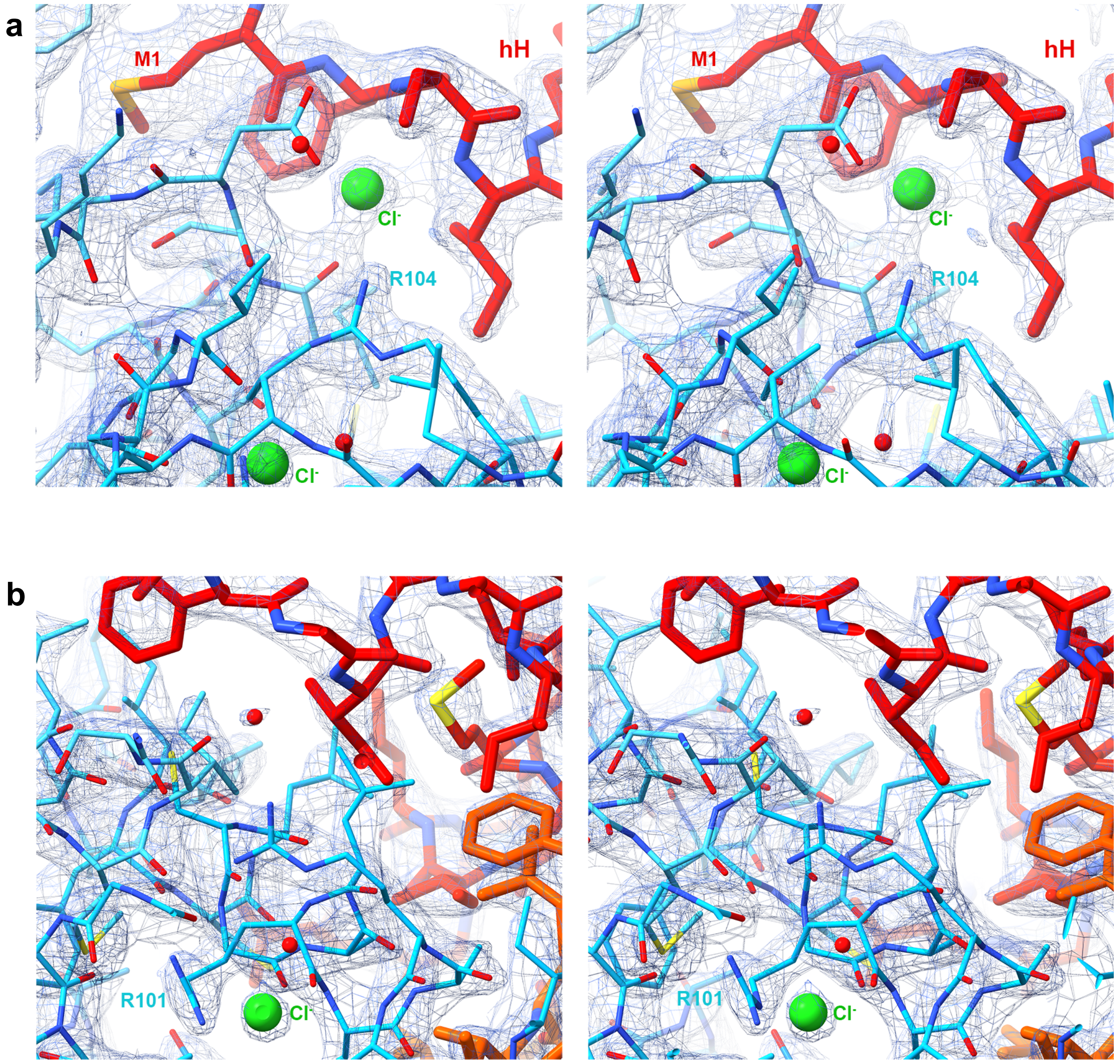

Supplementary Figure 6: Stereo representation of the electron density for the interface region of the N-terminal horizontal helix of one protomer (red) and helices 4 and 6 from the other protomer (blue) for the open (a) and closed (b) conformation. The two chloride ions (out of a total of wthree) in the open conformation are shown as green spheres. In the closed conformation, the electron density at the position of the third chloride ion is too weak and is modelled here as water (red sphere).

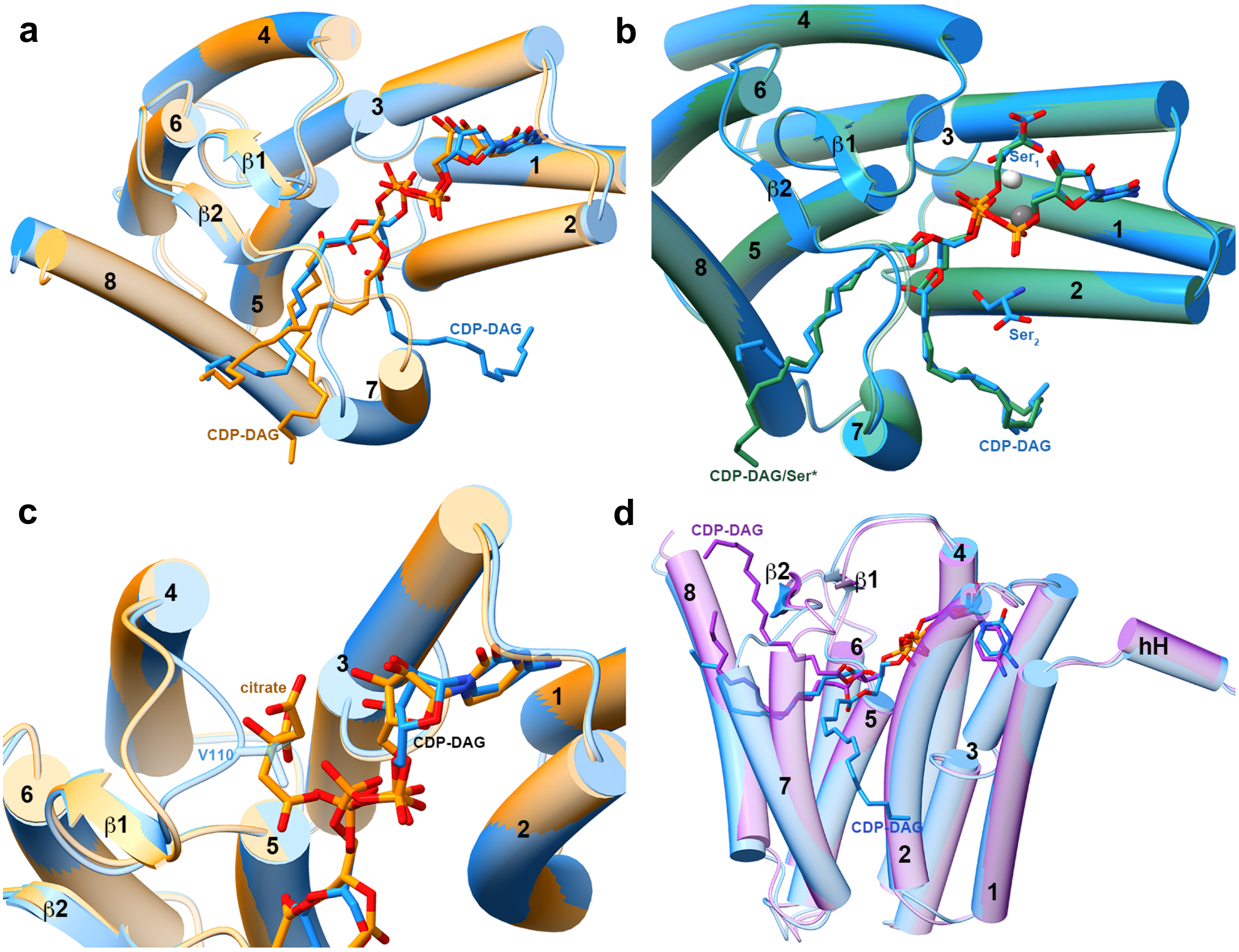

Supplementary Figure 7: Structural differences in MjPSS. a) Helix 7 is shifted towards helix 2 in the closed conformation (orange) and blocks the side path from the substrate binding site to the membrane core. In the open conformation (blue), helix 7 is shifted, opening the side path for one alkyl chain of CDP-DAG to the membrane. b) The overall conformation of the serine bound structures in the open conformation is largely identical. The main differences are the position of Ser_1, the presence of electron density between Ser_1 and CDP-DAG, and the coordination geometry of the β-phosphate, which is tetragonally coordinated in the structure with two serines (blue) and pentagonal coordinated in the other (green), indicating a transition state of the CDP-DAG/serine complex. c) The main difference between the two closed structures is found in the loop region between the C-terminus of helix 4 and β1. This loop is displaced in the closed conformation with bound citrate (orange), whereas in the closed conformation without citrate (blue) the position of citrate is occupied by Val110 from the loop. d) There are also clear differences in the conformation of the CDP-DAG alkyl chains. In one of the closed structures (purple) the alkyl chain ends point toward the cytoplasm, while in the open conformations one of the alkyl chain ends is closer to the other side of the membrane.

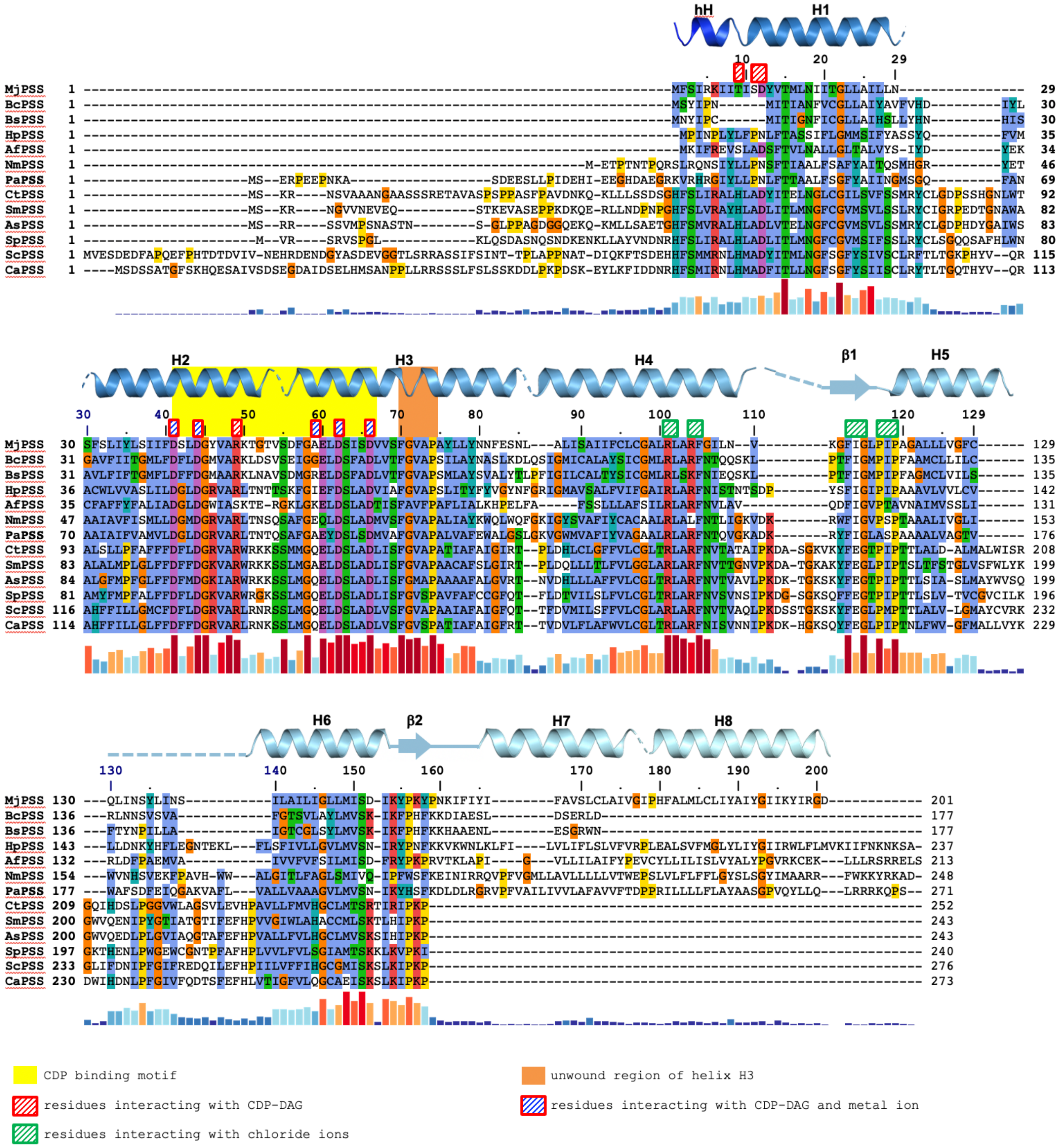

Supplementary Figure 8: Sequence alignment of phosphatidyl serine synthases (PSS) from different organisms. The secondary structure of MjPSS is shown above, and the sequence conservation is shown as colored bars below the sequence. The CDP-AP sequence motif and residues involved in serine, metal ion and chloride ion coordination are highlighted. The sequence numbers above the alignment correspond to MjPSS. The source organisms for the different PSS sequences in the alignment including the accession codes are listed below:

MjPSS: *Methanocaldococcus jannaschii* (Q58609.1)
BcPSS: *Bacillus cereus* (WP_071732650.1)
BsPSS: *Bacillus subtilis* (KIX81455.1)
HpPSS: *Helicobacter pylori* (AAC45587.1)
AfPSS: *Archaeoglobus fulgidus* (HGQ79278.1)

NmPSS: *Neisseria meningitidis* (NP_274337.1)
PaPSS: *Pseudomonas aeruginosa* (NP_253381.1)
CtPSS: *Chaetomium thermophilum* (XP_006695244.1)
SmPSS: *Sphaerulina musiva* (XP_016756997.1)
AsPSS: *Aspergillus flavus* (QRD89152.1)
SpPSS: *Schizosaccharomyces pombe* (NP_588326.2)
ScPSS: *Saccharomyces cerevisiae* (NP_010943.3)
CaPSS: *Candida albicans* (XP_713810.2)

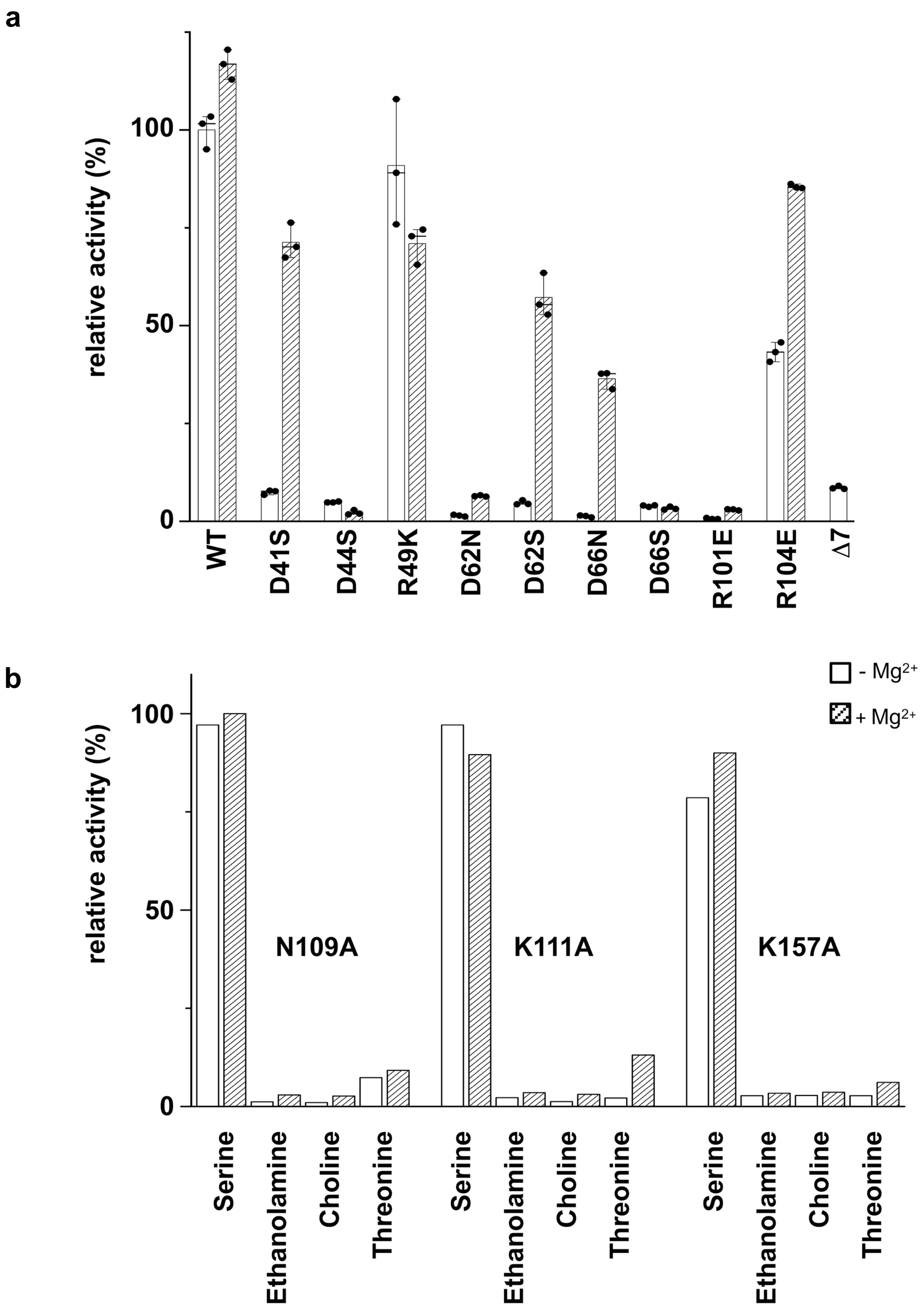

Supplementary Figure 9: Activity of MjPSS mutants. a) Relative enzyme activity of selected MjPSS mutants in the presence and absence of Mg^{2+} (n = 3; mean ± s.d.). b) The substrate selectivity of MjPSS is not altered by mutation of the residues involved in coordination of citrate and the second serine.

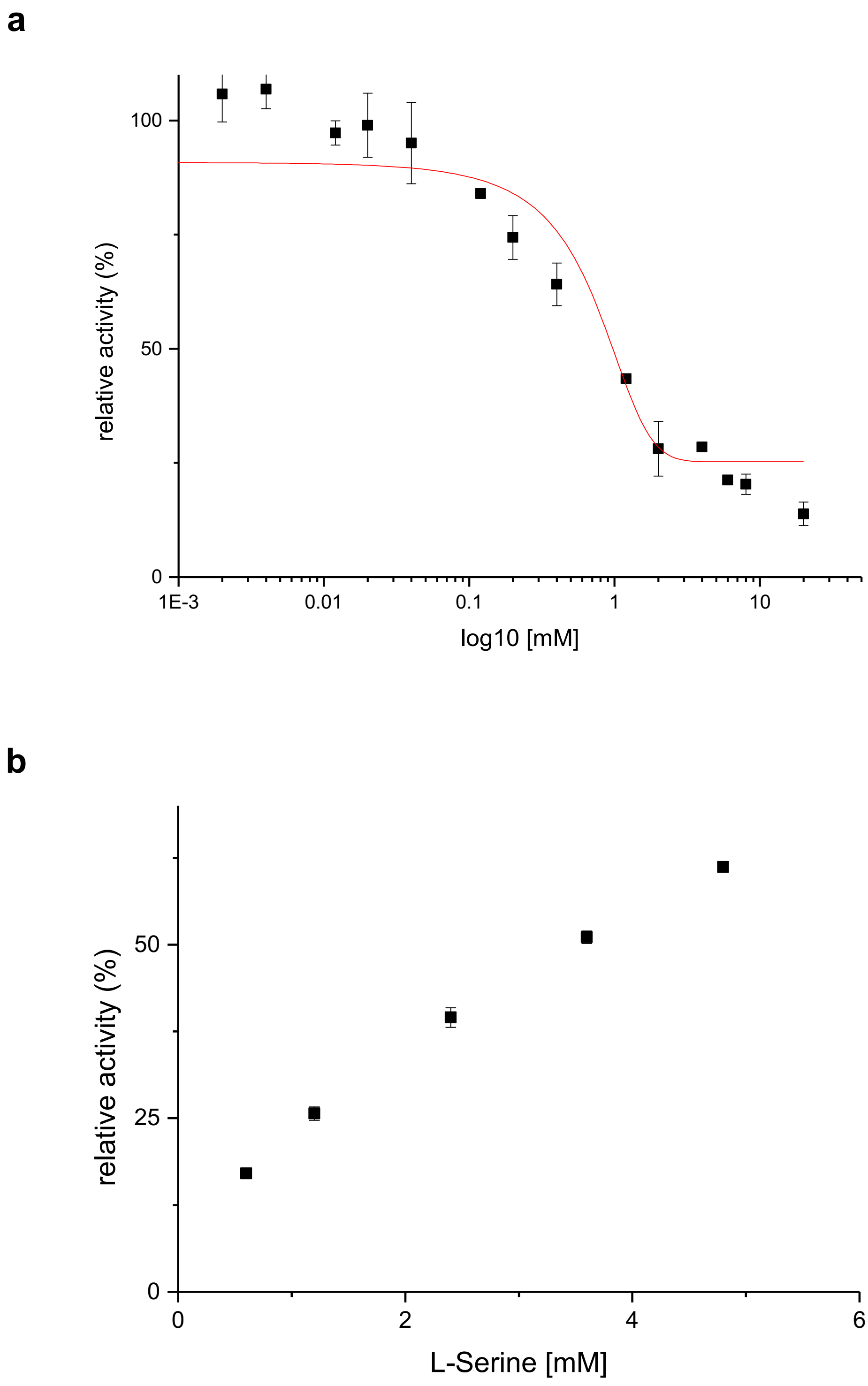

Supplementary Figure 10: Competition between citrate and serine in MjPSS. a) Inhibition of MjPSS by citrate was measured by adding citrate to the reaction mixture and measuring CMP release by HPLC. The IC50 for citrate is 4.1 ± 1.4 mM. Inhibition at higher citrate concentration is not complete as there is still residual activity of 20% (n = 3; mean ± s.d.). b) The activity is restored by adding of serine to the reaction mixture after inhibition with citrate (n = 3; mean ± s.d.).

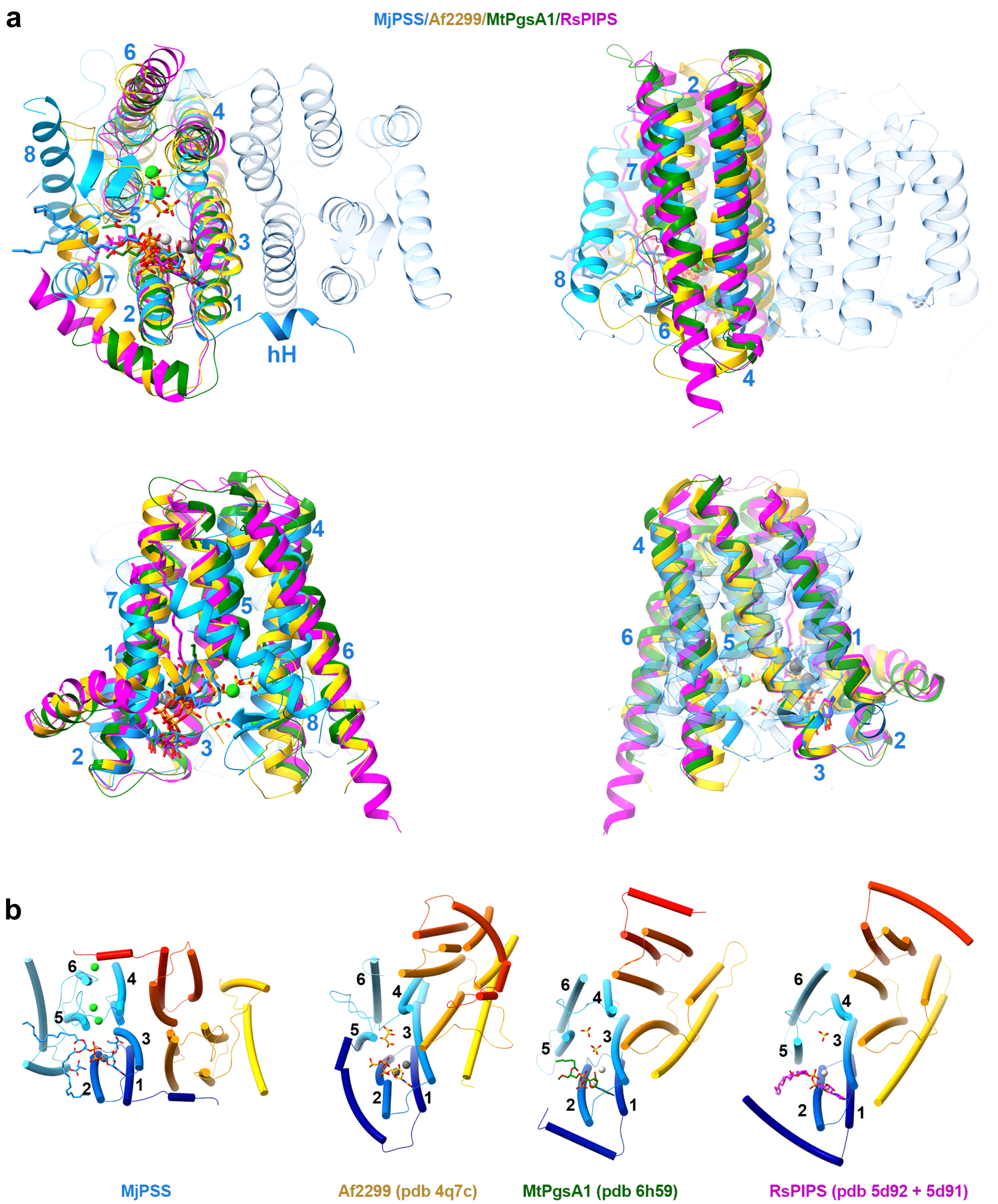

Supplementary Figure 11: Structural comparison of MjPSS with the phosphatidylinositol phosphate synthases Af2299, MtPgsA1, and RsPIPS. a) Superposition of all structures to one MjPSS monomer show that the two additional transmembrane helices of MjPSS have no counterparts in the other structures with six transmembrane helices. All structures have an additional helix of variable length in the N-terminus. The N-terminal helix in MjPSS is oriented in the opposite direction to the second monomer, whereas the corresponding helices in the other structures point to the opposite direction and partially obscure the substrate binding site. b) The relative orientation of the protomers within the compared dimers. In MjPSS, helix 1, 3 and 4 of each protomer form the dimer interface. Helix 1 is not involved in dimer formation in Af2299,

MtPgsA1 and RsPIPS. The dimers are aligned by superposition of helices 1-6 of a single protomer (blue). The bound substrates are shown as sticks with the same color code as above. In RsPIPS, the sulphate molecule is superimposed from pdb 5d91 into pdb 5d92.

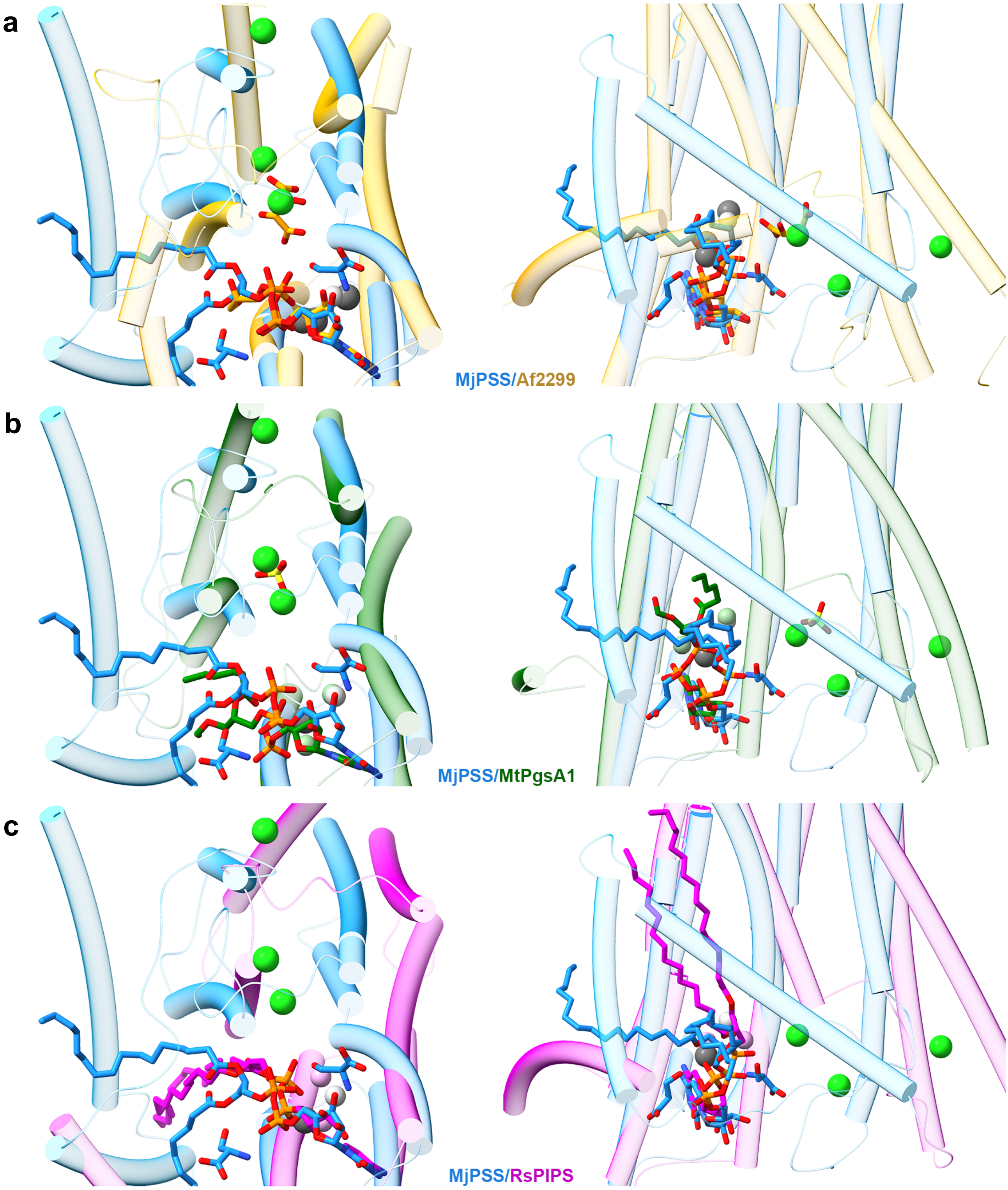

Supplementary Figure 12: The individual comparison of MjPSS with other CDP-APs, viewed from two different angles. a) In Af2299, a tartrate molecule is in the same position as one of the chloride ions in MjPSS. b) The position of the CDP moiety in PgsA1 is the same as that of CDP-DAG in MjPSS, but the CDP-DAG in MtPgsA1 is modeled as CDP-glycerol because the alkyl chains are not resolved, probably due to higher flexibility. MtPgsA1 has two sulfate ion binding sites, one near the position of the serine and one of the chlorides in MjPSS. c) RsPIPS has only one sulfate near the position of chloride in MjPSS. The bound CDP-DAG in PsPIPS is resolved, but its alkyl chains are orthogonal to that of MjPSS.

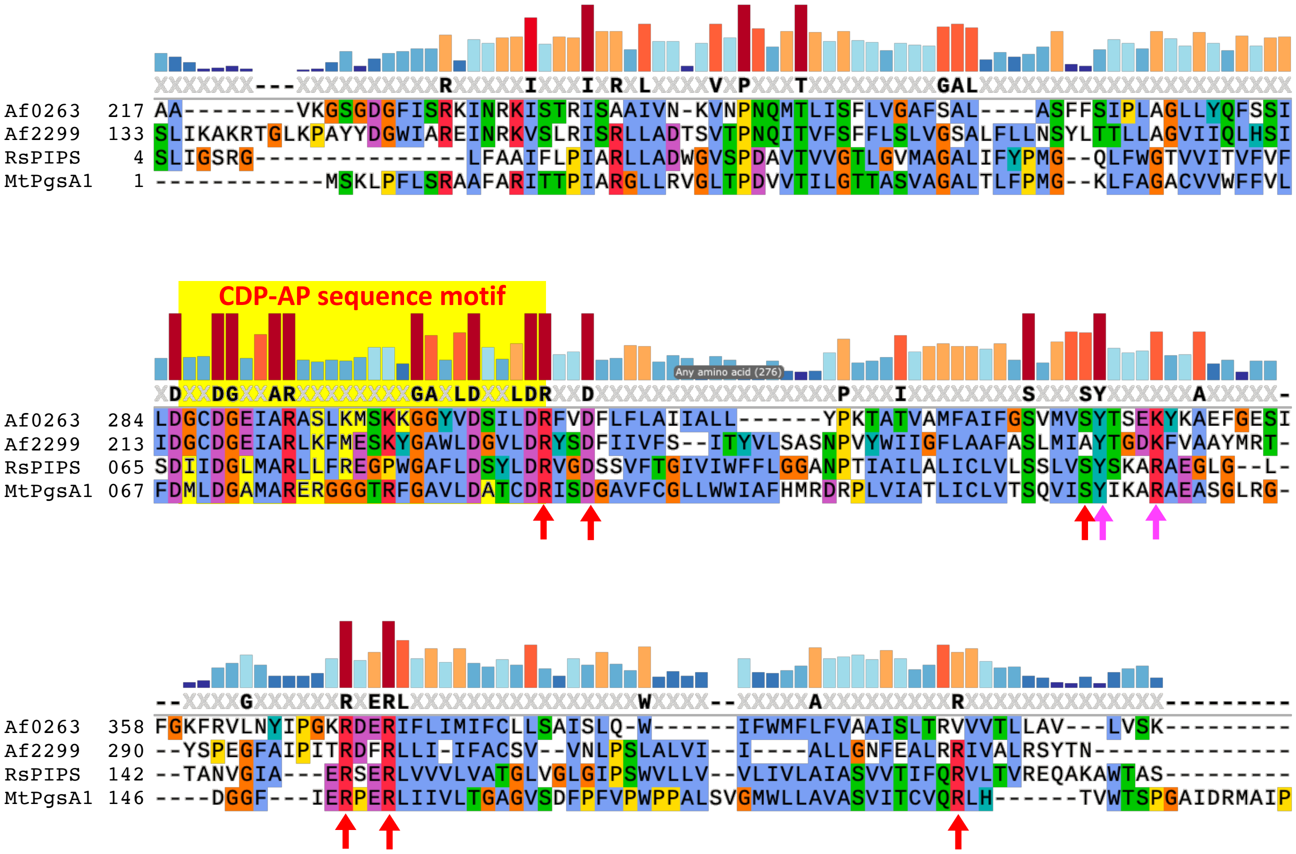

Supplementary Figure 13: Sequence alignment of CDP-APs with known structure. Sequence conservation is shown as coloured bars above the sequence. The CDP-AP sequence motif is highlighted in yellow. Residues involved in anion coordination are indicated by arrows below the sequence alignment. The purple arrows indicate the anion coordinating residues from the second monomer.

MjPSS_D41S_F	5´ CCTCTCAATAATCTTTTCATCTTTAGATGGATATGTAGCAAG 3´
MjPSS_D41S_R	5´ GCTACATATCCATCTAAAGATGAAAAGATTATTGAGAGGTAG 3´
MjPSS_D44S_F	5´ CAATAATCTTTGATTCTTTATCAGATATGTAGCAAGAAA 3´
MjPSS_D44S_R	5´ GTTTTTCTTGCTACATATCTGATAAAGAATCAAAGATTA 3´
MjPSS_R49K_F	5´ TAGATGGATATGTAGCAAACAAAACTGGAACTGTCTC 3´
MjPSS_R49K_R	5´ ACAGTTCCAGTTTTGTTTGCTACATATCCATCTAAAG 3´
MjPSS_D62S_F	5´ TTGGGGCTGAGTTATCAAGTATTTCAGATGTAGTTAGC 3´
MjPSS_D62S_R	5´ AACTACATCTGAAATACTTGATAACTCAGCCCCAAAGT 3´
MjPSS_D66S_F	5´ GGGGCTGAGTTAGACAGTATTTCATCAGTAGTTAGCTTTGGAGTAGC 3´
MjPSS_D66S_R	5´ GGAGCTACTCCAAAGCTAACTACTGATGAAATACTGTCTAACTCAGC 3´
MjPSS_D62N_F	5´ CCGATTTTGGTGCTGAACTGAATAGCATTAGTGACGTTG 3´
MjPSS_D62N_R	5´ CAACGTCACTAATGCTATTCAGTTCAGCACCAAAATCGG 3´
MjPSS_D66N_F	5´ CTGAACTGGATAGCATTAGTAACGTTGTGTCGTTTGGCG 3´
MjPSS_D66N_R	5´ CGCCAAACGACACAACGTTACTAATGCTATCCAGTTCAG 3´
MjPSS_R101E_F	5´ TGTGCGGTGCCCTGGAATTAGCGAGATTTGGCATTTTAAACGTC 3´
MjPSS_R101E_R	5´ GACGTTTAAAATGCCAAATCTCGCTAATTCCAGGGCACCGCACA 3´
MjPSS_R104E_F	5´ TGTGCGGTGCCCTGCGTTTAGCGGAATTTGGCATTTTAAACGTC 3´
MjPSS_R104E_R	5´ GACGTTTAAAATGCCAAATTCCGCTAAACGCAGGGCACCGCACA 3´
MjPSS_R101/104E_ F	5´ TGTGCGGTGCCCTGGAATTAGCGGAATTTGGCATTTTAAACGTC 3´
MjPSS_R101/104E_ R	5´ GACGTTTAAAATGCCAAATTCCGCTAATTCCAGGGCACCGCACA 3´
$MjPSS_{\Delta 1\text{-}8}$_F	5´ ATCCATATGACTATCAGCGACTATGTGACC 3´
$MjPSS_{\Delta 1\text{-}8}$_R	5´ CTCGACTTACTCGAGATCGCCACGGATATAC 3´

Supplementary Table 1: Oligonucleotides used for site-directed mutagenesis.
